# Lung Abscess in a Patient With VAP: A Rare Case of Lung Infection Complicated by Two Pathogens

**DOI:** 10.4021/jocmr1132w

**Published:** 2013-01-11

**Authors:** Christina Mystakelli, Stavros Gourgiotis, Paraskevi Aravosita, Charalampos Seretis, Efthymia Kanna, Stavros Aloizos

**Affiliations:** a Intensive Care Unit, “Mitera” Obstetric and Gynecological Hospital, Athens, Greece

**Keywords:** Ventilator-associated pneumonia, Acinetobacter baumannii, Klebsiella pneumoniae, Lung, Abscess

## Abstract

Ventilator-associated pneumonia (VAP) is defined as pneumonia occurring in a patient after intubation with an endotracheal tube or tracheostomy tube lasting for 48 hours or more. We describe a case of 75-year-old male who initially presented with pneumonia of the right basis with accompanying plevritis. The patient was intubated and his condition was complicated with a VAP infection while he developed a lung abscess. The antibiotic therapy was based on susceptibility bronchial secretions isolated acinetobacter baumannii and klebsiella pneumoniae; these pathogens were also isolated from the drained abscess. The patient was discharged in good health. The interest of this case is recommended in the existence of two responsible pathogens, the paucity of the development of lung abscess in a patient with VAP, and the successful treatment of the patient with the combination of controlled drainage of the abscess and appropriate antibiotic therapy.

## Introduction

Ventilator-associated pneumonia (VAP) is a common, possibly life-threatening complication in intensive care unit (ICU) patients with a reported relative risk of 9-27% and mortality of 25-50% [1].

The pathogenesis of VAP is supported to initiate from the aspiration of oropharyngeal secretions past the endotracheal tube cuff or from inoculation directly into the airway, resulting finally in the development of severe inflammation of the respiratory system [2].

VAP should be distinguished from the community-acquired pneumonia, which is related to acute respiratory failure, and from nosocomial pneumonia occurring among hospitalized patients, who, on the contrary, do not receive mechanical ventilation. According to CDC guidelines, the diagnosis of VAP in a patient receiving ventilation support are the presence of new and/or progressive pulmonary infiltrates on a chest radiograph plus two or more of the following criteria: fever (≥ 38.5 °C) or hypothermia (< 36 °C), leukocytosis (≥ 12 × 10^9^/L), purulent tracheobronchial secretions, or a reduction in PaO_2_/FIO_2_ of at least 15% in the previous 48 hours [3].

We present an extremely rare case of a patient with VAP who developed lung abscess, due to acinetobacter baumanii infection and who was treated with administration of mereponem and colistin combined with computed tomography (CT)-guided drainage. The presence of two main pathogens, the rarity of the development of a lung abscess in a patient with VAP, and the successful management with the combination of CT-guided drainage and cultures-based antibiotic therapy underline the diagnostic and educational value of this case.

## Case Report

**Figure 1:**
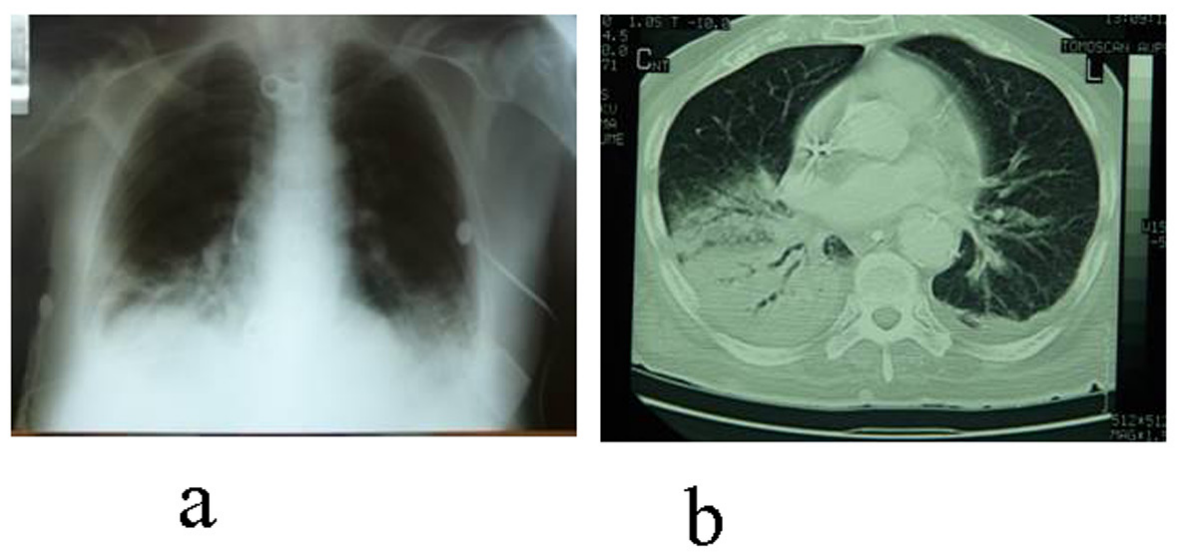
Chest X-ray (a) and CT (b) reveal pneumonia of the right basis with accompanying plevritis.

A 75-year-old male with a history of diabetes, heart failure and chronic obstructive pulmonary disease was admitted to hospital with a 2 days history of cough, fever (> 38 °C) and shortness of breath. Chest x-ray and chest CT revealed pneumonia of the right basis (Fig. 1). Under the diagnosis of community-acquired pneumonia, the administration of piperacillin-tazobactam (i.v. and nebulizer) was initially started. Nevertheless, the patient was intubated a few hours later due to hemodynamic instability and insufficient gas exchange. The antibiotic therapy was continued with no patient’s clinical improvement. Acinetobacter baumannii and klebsiella pneumoniae were isolated from the bronchial secretions and a new antibiotic i.v. scheme included meropenem and colistin was administrated; the scheme was based on his bronchial cultures susceptibility while the diagnosis of VAP was established.

**Figure 2:**
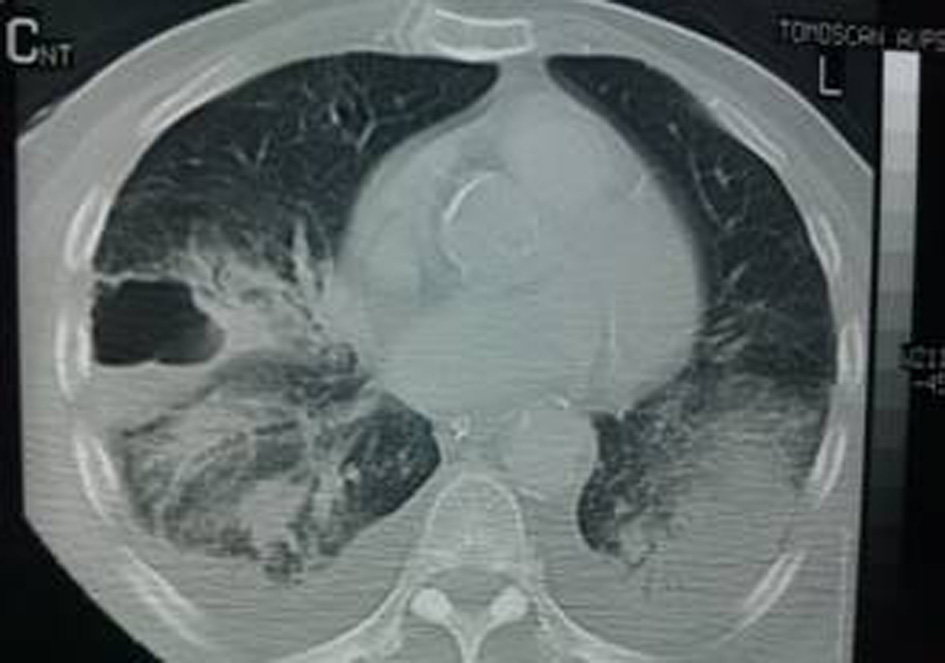
CT imaging of the lung abscess.

Fifteen days later, a new chest CT was performed due to patient’s afternoon fever (up to 38.2 °C) and the persistence of his symptoms. The CT revealed the existence of a 4.2 cm × 4 cm × 3.5 cm abscess in the middle lobe of the right lung (Fig. 2). CT-guided drainage of the abscess was decided. Acinetobacter baumannii and klebsiella pneumoniae were also isolated from the drained fluid. The existing antibiotic therapy was continued. The patient was discharged some days later in good health.

## Discussion

VAP is considered to have become a life-threatening situation in ICU patients. The main reasons are the increased resistance of nosocomeal pathogens in antibiotics administration and the impaired immune response of the critically ill patients.

The most common pathogens that are related to the development of VAP are pseudomonas aeruginosa (about 24%), staphylococcus aureus (about 20%), haemophilus species (about 10%), acinetobacter species (about 8%), streptococcus pneumoniae (about 4%) and klebsiella species (about 2%) [4]. The therapeutic approach of VAP remains a matter of debate; nevertheless, in the daily practice, the main targets are to prevent or confront the migration of the pathogens to the lower respiratory structures and eradicate the developing respiratory infection with the use of appropriate antibiotics. From this point of view, it is evident that the detection of the specific pathogen(s) that cause(s) VAP has a crucial role concerning the type of antibiotics that should be administered, both in order to confront the infection and not violate the principles of evidence-based use of antibiotics.

The most important factors towards this direction are the thorough clinical examination and the close monitoring of the patients, the avoidance of using antibiotics without evidence from antibiogrammes and the capability of multidisciplinary approach of the patients in complicated cases.

In the case presented, VAP was complicated by the formation of a lung abscess, induced by acinetobacter baumanii co-infection. The development of lung abscesses in VAP are rare complications, particularly coming to the presence of acinetobacter baumanii, with almost complete lack of similar case reports, whose rarity could be attributed to the extensive use of broad-spectrum antibiotics in these patients [5].

The therapeutical approach was very challenging. The antibiotics administrated, meropenem and colistin, were proven to be an efficient treatment strategy, managing to intercept the progress of a severe respiratory infection in a critically ill patient, in accordance to the literature [5, 6]. In addition, CT-guided drainage of the lung abscess, which followed in sequence, appeared to be an effective method to treat a lung abscess that was refractory to conventional therapy.

In conclusion, the successful management of this severe infection, along with its rare complication by the particular species, lied upon the combination of aggressive -but not excessive- antibiotics treatment in combination with interventional radiology applications, underlining the significance of multidisciplinary approach in rising up to the task of coping with complex and life-threatening infections in the ICU environment.
